# Early versus late 2 mg/kg methylprednisolone therapy in ARDS

**DOI:** 10.1038/s41598-025-22142-8

**Published:** 2025-10-31

**Authors:** Justine Verchère, Damien Barrau, Jonathan Chelly, Julien Carvelli, Lionel Velly, Nicolas Bruder, David Lagier, Antoine Bianchi, Christophe Guervilly, Anderson Loundou, Noémie Peres, Jean-Marie Forel

**Affiliations:** 1 Médecine Intensive et Réanimation, Hôpital Nord, Centre Hospitalier Universitaire, Assistance Publique Hôpitaux de Marseille, Chemin des Bourrely, 13915 Marseille, France; 2 Réanimation, Centre Hospitalier Intercommunal Toulon La Seyne sur Mer, Hôpital Sainte Musse, Toulon, France; 3 Médecine Intensive et Réanimation, Réanimation Des Urgences, Hôpital de la Timone, Centre Hospitalier Universitaire, Assistance Publique Hôpitaux de Marseille, 13005 Marseille, France; 4 Service d’anesthésie-Réanimation, Réanimation Polyvalente, Hôpital de La Timone, Centre Hospitalier Universitaire, Assistance Publique Hôpitaux de Marseille, 13005 Marseille, France; 5 Service d’anesthésie-Réanimation, Réanimation Polyvalente Et Centre Inter-Régional Méditerranée Grands Brûlés, Hôpital de La Conception Centre Hospitalier Universitaire, Assistance Publique Hôpitaux de Marseille, 13005 Marseille, France; 6 Service d’anesthésie-Réanimation, Réanimation Cardio Vasculaire, Hôpital de la Timone, Centre Hospitalier Universitaire, Assistance Publique Hôpitaux de Marseille, 13005 Marseille, France; 7 Service d’anesthésie-Réanimation, Réanimation Polyvalente et Centre de Traumatologie, Hôpital Nord, Centre Hospitalier Universitaire, Assistance Publique Hôpitaux de Marseille, Chemin des Bourrely, 13915 Marseille, France; 8 CEReSS–Centre d’Etudes et de Recherches Sur les Services de Santé et Qualité de Vie Aix-Marseille Université, Faculté Des Sciences Médicales Et Paramédicales- Campus La Timone, 3279 Marseille, EA France; 9 Support Unit for Clinical Research and Economic Evaluation, Department of Research and Innovation, Aix-Marseille University, 264, Rue Saint Pierre, 13385 Marseille, France; 10 GRAM+: Groupe de Recherche en Réanimation et Anesthésie de Marseille Pluridisciplinaire, Centre Hospitalier Universitaire, Assistance Publique Hôpitaux de Marseille, Marseille, France

**Keywords:** Methylprednisolone, Corticosteroid, Persistent ARDS, Late, Diseases, Medical research, Signs and symptoms

## Abstract

The fibroproliferative stage and persistent inflammation of acute respiratory distress syndrome (ARDS) are key factors leading to either the resolution of the syndrome or fibrosis. Previous studies suggest that a corticosteroid therapy promotes the evolution of ARDS toward an adapted repair process whereas others suggest that this therapy increases the risk of death if it starts more than 14 days after ARDS onset. Since the efficacy and safety of delayed 2 mg/kg methylprednisolone therapy in patients with ARDS is a matter of debate, we performed this observational multicentric retrospective study. We analysed the data of 392 patients with ARDS who received 2 mg/kg methylprednisolone therapy. The primary endpoint was mortality six months after 2 mg/kg methylprednisolone therapy was started. The secondary endpoints included mortality 60 days after the corticosteroid therapy initiation and the number of ventilator-free days (VFDs) and intensive care unit (ICU)-free days. We investigated the occurrence of complications such as ventilator-acquired pneumonia (VAP), septic shock and gastrointestinal bleeding arising after the start of the protocol. A total of 189 (48.2%) patients received 2 mg/kg methylprednisolone therapy within the first 14 days of ARDS onset. A total of 203 (51.8%) patients received it more than 14 days included post-ARDS-onset. The mortality rate six months after the initiation of 2 mg/kg methylprednisolone therapy was 51.9% in the early initiation group and 52.2% in the late initiation group (*p* = 0.942). The mortality rate 60 days after the initiation of 2 mg/kg methylprednisolone therapy was 47.1% in the early group and 47.3% in the late group (*p* = 0.968). There was no significant difference in the number of VFDs (*p* = 0.336) or ICU-free days (*p* = 0.175) 60 days after the start of the 2 mg/kg protocol. Initiating the protocol 14 days after the onset of ARDS seemed to be associated with more complications (*p* < 0.001). Late initiation was associated with greater occurrence of VAP (*p* = 0.018) or gastrointestinal bleeding (*p* = 0.012). These results suggest that an initiation of 2 mg/kg methylprednisolone therapy after 14 days from ARDS onset is not associated with an increased risk of death as compared with initiation prior to day 14. Delayed 2 mg/kg methylprednisolone therapy in patients with persistent ARDS should be considered.

## Introduction

Acute respiratory distress syndrome (ARDS) has a major epidemiological impact, with an estimated mortality rate estimated of 35–45%^1^. Surviving patients are impacted by respiratory failure, muscular weakness and anxiety-depressive disorders^2^. ARDS is characterized by inflammatory lung injury leading to hypoxemic respiratory failure. This inflammatory process causes alterations in the alveolar-capillary membrane, with impaired fluid resorption and accumulation of proteins and cells in the alveolar space. This syndrome impacts surfactant function and leads clinically to atelectasis, reduced ventilatory volumes, shunts and hypoxemia. Proinflammatory cytokines can also diffuse systemically, causing major vascular failure. Systemic inflammation induces glucocorticoid receptor resistance/decreased sensitivity, which can be countered by prolonged corticosteroid treatment^3^. This phase is quickly followed by a fibroproliferative phase, leading to an adapted repair process and the resolution of the syndrome or to the dysregulation of inflammation and subsequent fibrosis. Therefore, the fibroproliferative stage is key period. It is legitimate to think that an anti-inflammatory therapeutic action, such as corticosteroid therapy, could promote the evolution of the syndrome towards an adapted repair process. Identifying this key time is complex as a temporal criterion is not yet available.

Some authors, including Villar et al. and Meduri et al., advocate the concept of early immunomodulation in ARDS to improve survival and ventilator-free days (VFDs). In DEXA-ARDS, Villar et al. suggested that treatment with dexamethasone 20 mg IV (Eq. 1.5 mg/kg methylprednisolone) be initiated within the first 24 h^4^. In their study published in Chest in 2007, Meduri et al. suggested that treatment with 1 mg/kg methylprednisolone be initiated within the first 72 h^5^. Previously in their 1998 study^6^, Meduri et al., established a 2 mg/kg protocol for ARDS patients receiving mechanical ventilation for more than 7 days. They reported a significant effect on intensive care unit (ICU) survival and mechanical ventilation weaning, in favour of corticosteroid therapy. In addition, recent studies have shown improvements in survival in three more specific indications: sepsis-induced ARDS^7^, acute community-acquired pneumonia (CAP)^8^ and COVID-19 pneumonia-induced ARDS^9^.

Although corticosteroid therapy seems to improve ARDS patients outcomes if it is initiated initially (≤ 72h)^4^,^5^ or in the early phase of ARDS (< day 14)^6^, its safety 14 days after the onset of ARDS remains a matter of debate. Indeed, in their study published in 2006 in the NEJM^10^, Steinberg et al. reported higher 60-day and six-month mortality rates in the subgroup of patients receiving 2 mg/kg methylprednisolone therapy after 14 days of mechanical ventilation. The main side events related to delayed corticosteroid therapy are VAP, septic shock, gastro-intestinal bleeding, hyperglycaemia and neuromyopathy. Overall, the benefit/risk balance seemed to favour corticosteroid therapy with lower mortality rates on day 28 and more ICU-free days and VFDs.

In their new recommendations (conditional recommendation, moderate certainty of evidence), the American Thoracic Society and the Society of Critical Care Medicine (SCCM) suggest the use of corticosteroids for patients with ARDS^11^,^12^. However, the American Thoracic Society warn practitioners of a late initiation after 14 days of ARDS. The SCCM suggest a “low-to-moderate dose and late” corticosteroid therapy until day 21. These observations motivated our study. We aimed to compare the outcomes of patients with an initiation of a 2 mg/kg methylprednisolone therapy 14 days after ARDS onset and those with an initiation prior to day 14. We hypothesized that the outcomes for our two groups would be comparable, considering that corticosteroid therapy is effective during the exudative and fibroproliferative phases, that the 14-day threshold is arbitrary and that its side effects are minor. Our primary outcome was mortality six months after the first day of the patient’s 2 mg/kg methylprednisolone therapy. Our secondary endpoints were mortality, ventilator-free days and ICU-free days at day 60. We also investigated the occurrence of complications such as VAP, septic shock and gastrointestinal bleeding, after the protocol start.

## Materials and methods

### Study design and settings

This was an observational multicentric retrospective study. It was performed in eight medico-surgical ICUs of university hospitals (AP-HM, Marseille, France) and one medical ICU of a general hospital (Sainte-Musse, Toulon, France), corresponding to a total of 100 beds.

### Population

We analysed the records of patients over 18 years of age who presented ARDS criteria and who required mechanical ventilation from January 2016 to December 2023. Moreover, they must have received “low-to-moderate-dose” methylprednisolone therapy starting at 2 mg/kg/d from day 1 to day 14 of treatment, followed by 1 mg/kg/d from day 15 to day 21, 0.5 mg/kg/d from day 21 to day 28, 0.25 mg/kg/d on days 29 and 30 and 0.125 mg/kg/d on days 31 and 32. Meduri established this protocol in his 1998 study^6^. Patients were excluded if they presented pulmonary fibrosis responsible for preexisting chronic respiratory insufficiency, if the aetiology of ARDS was known to be corticosteroid responsive (amiodarone, bleomycin, other autoimmune pneumopathy), if they died within 24 h of initiation of the 2 mg/kg methylprednisolone therapy protocol, or if they or their families were against the use of their health data for research purposes.

### Timing

Given that this study relies on temporal criteria, it is essential to detail the diagnostic approach used to determine the onset of ARDS. For patients intubated upon admission, the Berlin criteria were applied^13^. For those who were not initially intubated, the diagnosis of ARDS was based on a threshold of high-flow nasal oxygen (HFNO)—either 50L/min or 30L/min combined with a minimum of 8 h per day of continuous positive airway pressure (CPAP) or non-invasive ventilation (NIV). We relied on the limits of the Berlin definition raised by the European Society of Intensive Care Medicine (ESICM) in their late guidelines on ARDS to establish those criteria^14^. The physiopathological age of ARDS at the time of the 2mg/kg methylprednisolone initiation was defined as the delay between ARDS diagnosis and the initiation of the 2 mg/kg methylprednisolone therapy. We compared two patient groups according to their physiopathological age of ARDS—timing of their 2 mg/kg methylprednisolone therapy initiation. The early group included patients with an initiation of the therapy within day 14 from ARDS onset. The late group included patients with an initiation of the therapy on or after day 14 from ARDS onset. Outcomes durations were measured from the first day of the patients 2 mg/kg methylprednisolone protocol. Indeed, defining endpoints from the first day of the 2 mg/kg methylprednisolone protocol was considered more clinically meaningful and methodologically coherent.

### Study outcomes

The primary endpoint was mortality six months after initiation of 2 mg/kg methylprednisolone therapy according to its initiation prior to or after day 14 included of ARDS onset. We also assessed mortality 60 days after protocol initiation. The other secondary endpoints were the numbers of VFD and ICU-free days 60 days after protocol initiation. We also aimed to assess the occurrence of complications arising after the initiation of 2 mg/kg methylprednisolone therapy (VAP, septic shock, gastrointestinal bleeding) according to our two early and late groups. The diagnoses of VAP, sepsis, and gastrointestinal bleeding were based on established diagnostic criteria and clinical judgment. Those complications were clinician-reported events: documented and coded in the patients’ ICU discharge summary.

### Data collection

We screened every patient from the institutional clinical database with the keywords “ARDS” and “MEDURI protocol” or “steroids 2 mg/kg”. We collected patient-specific parameters (age, sex, medical history), pathology (aetiology, initial SAPSII), non-specific management of ARDS (continuous neuromuscular blockers, prone positioning, ECMO, duration of mechanical ventilation), and previous corticosteroid therapy. We did not collect patients’ ventilatory parameters, practitioners applied protective mechanical ventilation following established recommendations. Medical records were carefully reviewed to find these data.

### Statistical analysis

Quantitative variables are expressed as the mean (± standard deviation) or median (interquartile range). Comparisons between groups were performed using the Mann–Whitney U test or Student’s t-test according to the distribution. The distribution was evaluated using the Shapiro–Wilk test. Qualitative variables are expressed as numbers (%). Missing data (0.8%) were addressed using a group-based imputation method with group medians. Comparisons between groups were performed using the chi-squared test or the Fisher’s exact test. First, we compared patient characteristics according to the timing of initiation of 2 mg/kg methylprednisolone therapy (within prior to or after day 14 included of ARDS onset). Second, we compared patient characteristics (including the timing of initiation of 2mg/kg methylprednisolone therapy initiation) according to six-month mortality, our primary outcome. A logistic regression multivariate analysis was subsequently performed to determine whether the timing of initiation prior to or after day 14 included of ARDS onset was independently associated with six-month mortality. Every variable with a *p* value < 0.20 in the univariate analysis was included in the model. Even if the difference was not significant in the univariate analysis, since COVID-19 patients and ECMO patients represent a specific population, we also added these two variables to the multivariate analysis. Multicollinearity was tested by calculating the variance inflation factors (VIF) before including the variables in the model. Calibration of the logistic model was assessed using the Hosmer–Lemeshow goodness-of-fit test to evaluate the discrepancy between the observed and expected values. Odds ratios were expressed with 95% confidence intervals. All the tests were two-sided, with statistical significance defined as *p* < 0.05. To better reflect the temporal dimension of ARDS pathophysiology, we performed an additional analysis incorporating the physiological age of ARDS at the time the 2 mg/kg methylprednisolone was initiated. We treated this delay as a continuous variable in the logistic regression model, rather than only with a binary classification (< 14 or ≥ 14 days). In addition to the multivariable logistic regression, we calculated the partial eta squared, following Cohen’s (1998) convention to estimate the effect size^15^. Statistical analysis was conducted using SPSS version 20.0 (NY, USA).

### Ethic

According to French law, as part of MR004, all participants were provided informed consent during study. A convention was signed between Assistance Publique–Hôpitaux Marseille (APHM) and Centre Hospitalier Intercommunal Toulon-La-Seyne-sur-Mer (Toulon Sainte-Musse) who authorized the treatment of its data by APHM. This study obtained the approval of the APHM Ethics and Scientific Committee N° CSE24-4 chaired by Pr. HARLE (cads@ap-hm.fr). All methods were performed in accordance with the relevant guidelines and regulations.

## Results

### Baseline characteristics

A total of 392 patients were included in the analysis. Baseline characteristics are shown in Table 1. We included mostly COVID-19 pneumonia (332 patients, 84.7%), bacterial pneumonia (25 patients, 6.4%) and influenza pneumonia (17 patients, 4.3%) patients. The median time between ARDS onset and initiation of the 2 mg/kg methylprednisolone protocol was 14 days [9–19]. Among the first 196 patients included (50%), the median delay between ARDS onset and 2 mg/kg methylprednisolone initiation was 15 days [10–19]. Among the last 196 patients included (50%), the median delay was 13 days [8–18]. The difference was not statistically significant (*p* = 0.425), suggesting that the distribution of early and late 2 mg/kg methylprednisolone initiation remained stable over the study period. The median time between mechanical ventilation and initiation of the 2 mg/kg protocol was 10.5 days [5–16]. We identified two groups: 189 patients (48.2%), who received 2 mg/kg methylprednisolone prior to day 14 of ARDS diagnosis and 203 patients (51.8%) who received 2 mg/kg methylprednisolone therapy after 14 days of ARDS diagnosis. There was a significant difference in age (*p* = 0.003), chronic immune suppression (*p* = 0.041), the mean SAPSII at admission (*p* = 0.044) and ECMO requirement (*p* < 0.001) between the early (< Day 14) and the late (≥ Day 14) groups.

**Table 1:** Baseline characteristics.

Characteristics	Overall = 392	< Day 14 (n = 189) *	≥ Day 14 (n = 203) *	*p*
Age (years)	61.7 (± 11.3)	63.5 (± 11.1)	60.1 (± 11.2)	0.003
Male sex	293 (74.7%)	139 (73.5%)	154 (75.9%)	0.598
Hypertension	177 (45.2%)	85 (45%)	92 (45.3%)	0.945
Chronic cardiac insufficiency	57 (14.5%)	30 (15.9%)	27 (13.3%)	0.470
Chronic renal insufficiency	12 (3.1%)	19 (10.1%)	11 (5.4%)	0.091
Chronic respiratory insufficiency	30 (7.7%)	5 (2.6%)	7 (3.4%)	0.773
Chronic immune suppression	96 (24.5%)	55 (29.1%)	41 (20.2%)	0.041
Diabetes	106 (27%)	50 (26.5%)	56 (27.6%)	0.801
SAPS II at admission	40 (± 12.2)	38.7 (± 10.9)	41.2 (± 13.2)	0.044
COVID19 ARDS	332 (84.7%)	163 (86.2%)	169 (83.2%)	0.483
Prone positioning**	392 (100%)	_	_	_
Neuromuscular blockers**	392 (100%)	_	_	_
ECMO**	148 (37.8%)	50 (26.5%)	98 (48.3%)	< 0.001
CS prior to the 2mg/kg MTP protocol	328 (83.7%)	161 (85.2%)	167 (82.3%)	0.495
No. of days between ARDS onset and the 2 mg/kg MTP protocol	14 [9–19]	8 [6–11]	18 [16–23]	< 0.001
No. of days between MV and the 2 mg/kg MTP protocol	10.5 [5–16]	5 [5–9]	16 [13–21]	< 0.001
ICU length of stay (days)	48.2 (± 31.1)	37.2 (± 27)	58 (± 31.3)	< 0.001

*SAPSII*, *simplified acute physiologic score; ECMO*, *extracorporeal membrane oxygenation; CS*, *corticosteroid; MTP*, *methylprednisolone; MV*, *mechanical ventilation.*

*189 patients received the 2 mg/kg methylprednisolone protocol within day 14, 203 after day 14 from ARDS onset. Results are represented as n (%) or means ± *SD or medians [IQR]. Because of rounding, percentages may not total 100. Immune suppression includes cancer and iatrogenic immunodepression.*

** During ICU stay: at least one session of 16h, 24h of neuromuscular blockers, ECMO.

### Overall population

#### Mortality

**Fig. 1:**
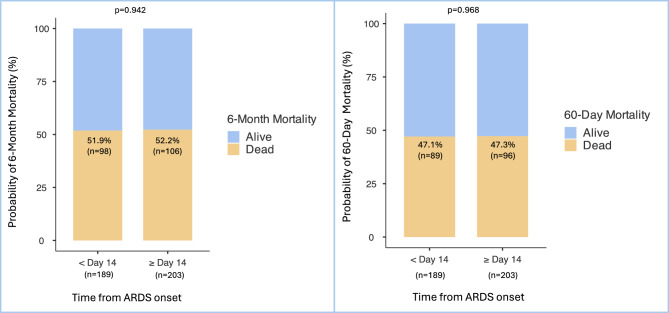
**A** 6-month mortality according to time from ARDS onset within or after day 14 (%). **B** 60-day mortality according to time from ARDS onset within or after day 14 (%).189 patients received the 2 mg/kg methylprednisolone protocol within day 14, 203 after day 14 from ARDS onset. Results are represented as n (%). Because of rounding, percentages may not total 100. For every endpoint, day 1 refers to the 2 mg/kg methylprednisolone therapy initiation.

The six-month mortality rate after the initiation of 2 mg/kg methylprednisolone therapy was 52% (204 patients). Late initiation of 2 mg/kg methylprednisolone therapy, 14 days included after ARDS onset, did not increase six-month mortality (*p* = 0.942). This result was consistent for 60-day mortality (*p* = 0.968). Six-month and 60-day mortality rates according to the initiation of the 2 mg/kg methylprednisolone protocol prior to or after day 14 of ARDS onset are shown in Fig. 1A and B.

#### Ventilator-free days

The median VFD was 0 [0–28]. There was no significant difference in the VFDs on day 60 according to the initiation of the 2 mg/kg methylprednisolone protocol prior to or after day 14 of ARDS onset (*p* = 0.336). These data are shown on Table 2. In the subgroup of surviving patients, a significant difference was observed (*p* = 0.014) with more VFDs in the early initiation group (43 [4–54]) as compared with the late initiation group (28 [0–45]).

**Table 2:** Secondary outcomes on the overall population within or after day 14 from ARDS onset.

Endpoints**	Overall (N = 392)	< Day 14 (N = 189) *	≥ Day 14 (N = 203) *	*p*
No. of ventilator-free days at day 60	0 [0–28]	0 [0–37.3]	0 [0–28]	0.336
No. of ICU-free days at day 60	0 [0–15.5]	0 [0–25.3]	0 [0–10]	0.175

*189 patients received the 2 mg/kg methylprednisolone protocol within day 14 and 203 after day 14 from ARDS onset. Results are represented as median [IQR].

**For every endpoint, day 1 refers to the 2 mg/kg methylprednisolone protocol initiation.

#### Intensive care unit-free days

The median number of ICU-free days was 0 [0–15.5]. There was no significant difference in the number of ICU-free days on day 60 according to the initiation of the 2 mg/kg methylprednisolone protocol prior to or after day 14 of ARDS onset (*p* = 0.175). These data are shown on Table 2. In the subgroup of surviving patients, a significant difference was observed (*p* = 0.005) with more ICU-free days in the early initiation group (36 [0–50]) as compared with the late initiation group (16 [0–38]).

#### Complications

**Fig. 2:**
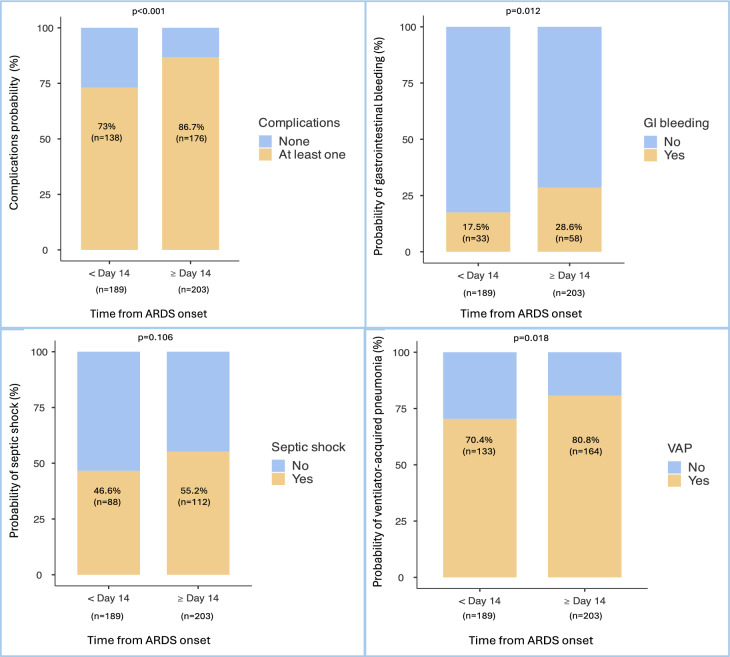
**A** At least one complication according to time from ARDS onset within or after day 14 (%), **B** Gastrointestinal bleeding according to time from ARDS onset within or after day 14 (%), **C** Septic shock according to time from ARDS onset within or after day (%), **D** Ventilator-acquired pneumonia according to time from ARDS onset within or after day 14 (%). 189 patients received the 2 mg/kg methylprednisolone protocol within day 14 and 203 after day 14 from ARDS onset. Complications arising after the 2 mg/kg protocol onset were collected. At least one complication = at least one VAP, septic shock or gastrointestinal bleeding, VAP = Ventilator-associated pneumonia, GI = Gastrointestinal bleeding

A total of 352 patients (89.8%) experienced at least one complication (VAP, new septic shock or gastrointestinal bleeding) after the initiation of the 2 mg/kg methylprednisolone therapy. As expected, the occurrence of at least one complication after protocol start was associated with the timing of initiation prior to or after day 14 of ARDS onset. Indeed, there were more patients with at least one complication in the late (≥ day 14) initiation group (*p* < 0.001) Fig. 2A. This result was driven mainly by the occurrence of gastrointestinal bleeding (*p* = 0.012) Fig. 2B and VAP (*p* = 0.018) Fig. 2D. In contrast, the occurrence of a new septic shock was not significantly different between the early and late groups (*p* = 0.106) Fig. 2C. Those results were not adjusted for confounding factors.

#### Multivariate analysis

The variables associated with six-month mortality, our primary endpoint, are shown in Tables 3 and 4.

**Table 3:** Variables associated with 6-month mortality: univariate and multivariate analysis.

	Alive (n = 188)	Dead (n = 204)	Univariate *p* value	Odd ratio	Multivariate *p* value	VIF
Age (years)	59 (± 11)	64 (± 11)	< 0.001	1.05 [1.03–1.08]	< 0.001	1.52
Chronic cardiac insufficiency	21 (11.2%)	36 (17.6%)	0.069	1.16 [0.61–2.22]	0.66	1.10
Hypertension	77 (41%)	100 (49%)	0.109	0.97 [0.60–1.56]	0.90	1.21
Diabetes	43 (22.9%)	63 (30.9%)	0.074	1.32 [0.78–2.25]	0.31	1.18
Chronic immune suppression	33 (17.6%)	63 (30.9%)	0.002	2.39 [1.39–4.11]	0.002	1.08
COVID-19 ARDS	164 (87.2%)	168 (82.4%)	0.180	0.85 [0.44–1.64]	0.62	1.14
SAPS II at admission	38 (± 12)	42 (± 13)	0.003	1.01 [0.99–1.03]	0.54	1.29
ECMO	68 (36.2%)	80 (39.2%)	0.534	1.45 [0.83–2.51]	0.19	1.49
Septic shock	70 (37.2.5%)	130 (63.7%)	< 0.001	3.33 [2.10–5.27]	< 0.001	1.12
2 mg/kg MTP initiation < day 14 from ARDS onset	91 (48.4%)	98 (48%)	0.942	1.08 [0.69–1.71]	0.73	1.09

Results are represented as n (%) or means ± SD. Because of rounding, percentages may not total 100. Multicollinearity was tested by calculating the variance inflation factors (VIF). Hosmer–Lemeshow goodness-of-fit test for the multivariate analysis demonstrated a Chi-square test score of 9.21 (p = 0.33). Immune suppression includes cancer and iatrogenic immunodepression.

MTP, methylprednisolone; SAPSII = simplified acute physiologic score; ECMO = extracorporeal membrane oxygenation; during ICU stay.

Septic shock arising after the 2 mg/kg protocol onset*.*

**Table 4:** Variables associated with 6—Month mortality: univariate and multivariate analysis.

	Alive (n = 188)	Dead (n = 204)	Univariate *p* value	Odd ratio	Multivariate *p* value	VIF
Age (years)	59 (± 11)	64 (± 11)	< 0.001	1.06 [1.03–1.08]	< 0.001	1.56
Chronic cardiac insufficiency	21 (11.2%)	36 (17.6%)	0.069	0.85 [0.44–1.63]	0.62	1.08
Hypertension	77 (41%)	100 (49%)	0.109	1.04 [0.65–1.69]	0.86	1.20
Diabetes	43 (22.9%)	63 (30.9%)	0.074	0.76 [0.45–1.3]	0.31	1.18
Chronic immune suppression	33 (17.6%)	63 (30.9%)	0.002	2.45 [1.42–4.21]	0.001	1.09
COVID-19 ARDS	164 (87.2%)	168 (82.4%)	0.180	1.18 [0.61–2.30]	0.63	1.12
SAPS II at admission	38 (± 12)	42 (± 13)	0.003	1.01 [0.99–1.03]	0.54	1.22
ECMO	68 (36.2%)	80 (39.2%)	0.534	1.40 [0.80–2.41]	0.25	1.51
Septic shock	70 (37.2.5%)	130 (63.7%)	< 0.001	3.30 [2.08–5.22]	< 0.001	1.12
Physiopathological age of ARDS at 2 mg/kg MTP initiation (days)	14.4 (± 9.66)	15.27 (± 9.19)	0.389	1.02 [0.99–1.044]	0.15	1.12

Results are represented as n (%) or means ± SD. Because of rounding, percentages may not total 100. Multicollinearity was tested by calculating the variance inflation factors (VIF). Hosmer–Lemeshow goodness-of-fit test for the multivariate analysis demonstrated a Chi-square test score of 13.62 (p = 0.092). Immune suppression includes cancer and iatrogenic immunodepression.

MTP, methylprednisolone; SAPSII, simplified acute physiologic score; ECMO, extracorporeal membrane oxygenation; during ICU stay.

Septic shock arising after the 2 mg/kg protocol onset.

Given that age, chronic cardiac insufficiency, hypertension, diabetes, chronic immunosuppression, SAPS II at admission, and septic shock seemed to be associated with mortality (*p* < 0.20), we performed a multivariate analysis including these covariates as a valuable method. Additionally, because COVID-19 and ECMO patients constitute specific populations, these two variables were also incorporated in the multivariate analysis. The variable: “timing of initiation of 2 mg/kg methylprednisolone therapy prior to or after day 14 of ARDS onset” was not associated with increased mortality (*p* = 0.942). This result was confirmed by the multivariate analysis (*p* = 0.73) (Table 3). The variable: “physiopathological age of ARDS at the time the 2 mg/kg methylprednisolone therapy was initiated”, taken as a continuous variable, was not associated with increased mortality (*p* = 0.389). This result was confirmed by the multivariate analysis (*p* = 0.15) (Table 4). Age, chronic immunosuppression and septic shock arising after the initiation of 2 mg/kg methylprednisolone therapy were associated with increased mortality. Indeed, according to Cohen’s (1998) convention^15^, the partial eta squared values indicated a moderate effect for age (0.046), a small to moderate effect for immunosuppression (0.026), and a moderate to large effect for septic shock (0.071).

### Non-COVID-19 patients

**Fig. 3:**
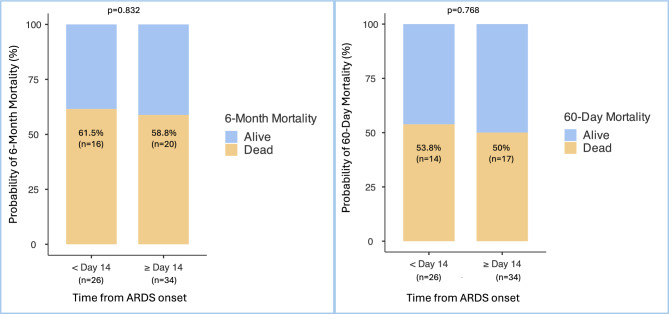
**A** 6-Month mortality on the non-COVID-19 patients according to time from ARDS onset within or after day 14 (%), **B** 60-Day mortality on the non-COVID-19 patients according to time from ARDS onset within or after day 14 (%), 26 patients received the 2 mg/kg methylprednisolone protocol within day 14, 34 after day 14 from ARDS onset. Results are represented as n (%). Because of rounding, percentages may not total 100. For every endpoint, day 1 refers to the 2 mg/kg methylprednisolone therapy initiation.

Our cohort included mostly COVID-19-related ARDS patients. One may wonder if our results are consistent in the non-COVID-19 population. We analysed 60 non-COVID-19 patients (15.3%). As in our overall population, six-month and 60-day mortality rates did not significantly differ between the two groups treated prior to or after day 14 of ARDS onset as shown in Fig. 3A and B. VFD and the number of ICU-free days on Day 60 in the non-COVID-19 population were not significantly different according to the timing of initiation of the 2 mg/kg methylprednisolone protocol prior to or after day 14 of ARDS onset (Table 5).

**Table 5:** Secondary outcomes on the non-COVID-19 patients within or after day 14 from ARDS onset.

Endpoints**	Overall (n = 60)	< Day 14 (n = 26) *	≥ Day 14 (n = 34) *	*p*
No. of ventilator-free days at day 60	0 [0–3]	0 [0–2]	0 [0–2.2]	0.760
No. of ICU-free days at day 60	0 [0–1.7]	0 [0–0]	0 [0–15]	0.873

*26 patients received the 2 mg/kg methylprednisolone protocol within day 14, 34 after day 14 from ARDS onset. Results are represented as medians [IQR].

**For every endpoint, day 1 refers to the 2 mg/kg methylprednisolone protocol initiation.

### Non-ECMO patients

**Fig. 4:**
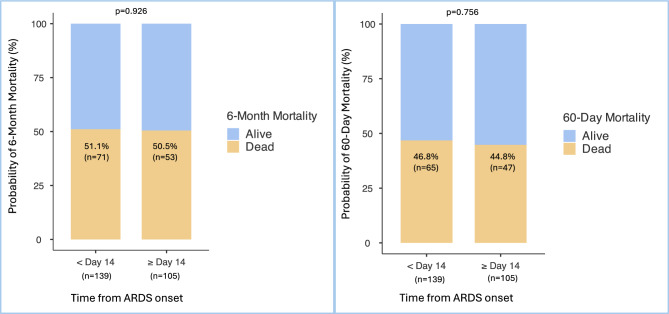
**A** 6-Month mortality on the non-ECMO patients according to time from ARDS onset within or after day 14 (%), **B** 60-Day mortality on the non-ECMO patients according to time from ARDS onset within or after day 14 (%). 139 patients received the 2 mg/kg methylprednisolone protocol within day 14, 105 after day 14 from ARDS onset. Results are represented as n (%). Because of rounding, percentages may not total 100. For every endpoint, day 1 refers to the 2 mg/kg methylprednisolone therapy initiation.

Our cohort included 148 patients (37.8%) assisted by venovenous ECMO. We analysed 244 non-ECMO patients (62.2%). As in our overall population, six-month and 60-day mortality rates did not significantly differ between the two groups treated prior to or after day 14 of ARDS onset as shown in Fig. 4A and B. VFD and the number of ICU-free days on Day 60 in the non-ECMO population were not significantly different according to the timing of initiation of the 2 mg/kg methylprednisolone protocol prior to or after day 14 of ARDS onset (Table 6).

**Table 6:** Secondary outcomes on the non-ECMO patients within or after day 14 from ARDS onset.

Endpoints**	Overall (n = 244)	< Day 14 (n = 139) *	≥ Day 14 (n = 105)*	*p*
No. of ventilator-free days at day 60	0 [0–42]	0 [0–45]	0 [0–37]	0.295
No. of ICU-free days at day 60	0 [0–35.5]	0 [0–41.3]	0 [0–20.5]	0.125

*139 patients received the 2 mg/kg methylprednisolone protocol within day 14, 105 after day 14 from ARDS onset. Results are represented as medians [IQR].

**For every endpoint, day 1 refers to the 2 mg/kg methylprednisolone protocol initiation.

## Discussion

In this retrospective study of 392 patients with ARDS, we report that the initiation of 2 mg/kg methylprednisolone therapy after 14 days from ARDS onset did not increase mortality as compared with initiation prior to day 14. There was no significant difference in six-month mortality after the start of the protocol according to the initiation of the 2 mg/kg methylprednisolone protocol prior to or after day 14 of ARDS onset (51.9% vs. 52.2%; *p* = 0.942). This result was also observed in the non-COVID-19 ARDS patients and the non-ECMO patients. The number of VFDs and ICU-free days 60 days after the start of the protocol were similar between the early (< day 14) and late (≥ day 14) initiation groups. Nevertheless, the outcomes VFDs and ICU-free days raise methodological issues since patients either stay in for a very long time or die. Thus, further analyses were conducted in the subgroup of surviving patients and a significant difference was observed. Therefore, a late initiation (≥ 14 days from ARDS onset) might delay mechanical ventilation weaning and ICU discharge without impacting mortality.

The percentage of patients with at least one complication including VAP, septic shock and gastrointestinal bleeding, arising after the start of the protocol, was greater in the late initiation group (73% vs. 86.7%; *p* < 0.001). We cannot determine if these complications were the consequence of a late initiation of the 2 mg/kg methylprednisolone protocol after day 14, or if it was because of a prolonged ICU length of stay. Indeed, the ICU length of stay was longer in the group of patients who received the protocol more than 14 days after ARDS onset. These patients were therefore more likely to present with complications. Although mortality was neutral, late therapy carried significantly more VAP and gastrointestinal bleeding; this trade-off deserves deeper discussion. A late initiation (≥ day 14), seemed to be associated with more VAP whereas septic shock was not. One may assume that this complication is well-known, diagnosed and treated. Therefore, it may delay ventilatory weaning without increasing mortality. In ARDS patients, Forel et al. did not find any association between VAP and ICU mortality after adjustment^16^. Headley et al. reported that appropriately treated nosocomial infections do not themselves cause death^17^. Gastrointestinal bleeding also seemed to increase with late corticosteroid initiation. A total of 148 patients (37.8%) were under venovenous ECMO and thus were likely under curative anticoagulation. We conducted further analysis in the non-ECMO population, and the difference in gastrointestinal bleeding was not significant between the two groups.

We enrolled patients between 2016 and 2023. Since 2020, the emergence of COVID-19-related ARDS has increased. Our cohort included mostly COVID-19-related ARDS patients. Furthermore, our study counted 148 (37.8%) very severe patients who required venovenous ECMO. It gave power to our study by allowing the inclusion of 392 patients. But one may wonder if our results are consistent in non-COVID-19 patients and non-ECMO patients. Thus, we analysed six-month mortality, VFDs and ICU-free days on day 60 among non-COVID-19 patients and non-ECMO patients. Among the non-COVID-19 patients (n = 60), a late initiation (≥ Day 14 of ARDS onset) of the 2 mg/kg methylprednisolone protocol did not increase six-month mortality or reduce VFD or ICU-free days at day 60. Similarly, among the non-ECMO patients (n = 244), late initiation (≥ Day 14 of ARDS onset) did not increase six-month mortality or reduce VFD or ICU-free days at day 60. Moreover, COVID-19 and ECMO variables were included in our multivariate analysis to reinforce those results.

The SCCM suggests 2 mg/kg methylprednisolone therapy until day 21, while the American Thoracic Society warned of a late initiation after day 14 of ARDS onset^11^,^12^. This warning mainly relies on Steinberg et al. study which provides a counterpoint to previously reported benefits of corticosteroid therapy^10^. In the overall population, the authors did not find a statistically significant reduction in 60-day mortality their primary outcome. Furthermore, the study identified potential adverse outcomes in a specific subgroup: methylprednisolone was associated with significantly increased 60-day and six-month mortality rates among patients enrolled at least 14 days after ARDS onset. Unlike in Steinberg’s trial, we used, as a temporal criterion, ARDS duration and not mechanical ventilation duration. We took into account the limits of a definition only based on the duration of mechanical ventilation which excludes patients under HFNO, as described in the guidelines of the ESICM^14^. In our study, the diagnosis of ARDS was based on a threshold of high-flow nasal oxygen (HFNO)—either 50L/min or 30L/min combined with a minimum of 8 h per day of continuous positive airway pressure (CPAP) or non-invasive ventilation (NIV). We believe this enhances the external validity of our study by aligning with routine clinical practice. Nevertheless, all patients required mechanical ventilation during their follow-up. In additional analyses (not shown), our results were the same when the timing of initiation of 2 mg/kg methylprednisolone therapy was based on the duration of invasive mechanical ventilation instead of the duration of ARDS. We did not find any significant difference in mortality, number of VFDs or number of ICU-free days according to this initiation time.

Our study has several strengths. First, it is a large cohort of 392 patients who received 2 mg/kg methylprednisolone therapy, an uncommon therapy. To our knowledge, the timing of initiation of corticosteroid therapy in ARDS patients is not clearly defined. We believe that our study provides new data on delayed corticosteroid therapy regardless of whether patients have received an early dose of corticosteroids. The most recent data recommend early treatment within 3 days of ARDS onset with a “low-to-moderate dose” (0.5–2 mg/kg eq. methylprednisolone)^4^,^5^ whereas recent studies and guidelines do not encourage initiation of a 2 mg/kg methylprednisolone therapy after day 14^10^,^11^. This was a multicentre study within several ICUs of university and general hospitals from southern France. The primary outcome was six-month mortality after initiation of the 2 mg/kg methylprednisolone protocol. Therefore, it captured vital status over an extended period. We were able to identify two initiation groups of patients according to the time of initiation of the therapy prior to or after day 14 of ARDS onset. The distributions of the patients in those two groups were symmetrical.

This study has also several limitations. Indeed, it was a retrospective study; even if patients’ medical records were carefully reviewed, every bias and specificity could not be avoided. First, clinically important differences appeared between our two initiation groups which warranted our multivariable model. Indeed, the late initiation group seemed to have more adverse baseline characteristics. However, this would typically suggest a higher mortality rate; interestingly, outcomes were comparable between the groups. We believe this strengthens our primary hypothesis: initiation of 2 mg/kg methylprednisolone therapy after day 14 of ARDS onset does not increase six-month mortality compared with that prior to day 14. In addition to this, we did not manage to collect severity scores or ventilatory parameters at the time 2 mg/kg methylprednisolone therapy was initiated. Furthermore, the lack of sample size / power analysis is a limitation that should be acknowledged. Although no formal post-hoc power analysis was performed, statistically significant differences were detected for several comparisons, indicating adequate sensitivity to detect medium-to-large effects. In their study, Steinberg et al. reported increased mortality in the subgroup of patients who initiated methylprednisolone therapy after 14 days. However, this observation emerged from a post-hoc analysis and was based on a limited sample size. Of the 89 patients treated with methylprednisolone, only 23 received late initiation (≥ 14 days), while 66 received early initiation (< 14 days). Despite a possible type II error, this study remains, to our knowledge, the largest to investigate this hypothesis. To improve the precision of our analysis and better account for the physiopathological age of ARDS, we performed an additional analysis using the delay between ARDS onset and 2 mg/kg methylprednisolone therapy initiation as a continuous variable, rather than a binary classification (< 14 days or ≥ 14 days). Nevertheless, the physiopathological age of ARDS at the time of initiation of 2 mg/kg methylprednisolone therapy, considered as a continuous variable, was not significantly associated with increased mortality. It is important to emphasize that we do not endorse delayed initiation of 2 mg/kg methylprednisolone therapy. Rather, our data suggest that, in cases where early administration is not possible, late initiation is not associated with increased mortality. Moreover, every patient received 2 mg/kg methylprednisolone therapy, thus our study lacked a control arm. We were not able to evaluate neuromyopathy because all our patients received the protocol and the intensity of the impairment was unknown, even if it is an adverse event usually associated with corticosteroid therapy, ICU-hospitalization, mechanical ventilation and even more when associated with neuromuscular blockers. Another well-known complication is increased glucose levels in patients receiving corticosteroid therapy. We were unable to assess the impact of the timing of initiation on this adverse event as it has rarely been reported in ICU reports. As already described, it included a great effective of COVID-19 and ECMO patients, although the results are consistent among non-COVID-19 patients and non-ECMO patients.

## Conclusion

In this large retrospective study, we have shown that the initiation of 2 mg/kg methylprednisolone therapy after day 14 of ARDS onset did not increase six-month mortality compared with that prior to day 14. This result should be confirmed with other prospective studies. Delayed initiation of 2 mg/kg methylprednisolone therapy in patients with persistent ARDS, notably when patients did not receive or do not improve after an early or less than 2 mg/kg eq. dose of methylprednisolone, can be considered.

## Data Availability

The datasets used and/or analyzed during the current study are available from the corresponding author on reasonable request.

## Abbreviations

APHM: Assistance publique hôpitaux marseillais
ARDS: Acuterespiratory distress syndrome
CPAP: Continuous positive airway pressure
CS: Corticosteroid
ECMO: Extracorporeal membrane of oxygenation
ESICM: European society of intensive care medicine
GI: Gastrointestinal bleeding
HFNO: High-flow nasal oxygen
ICU: Intensive care unit
MTP: Methylprednisolone
MV: Mechanical ventilation
NIV: Non-invasive ventilation
SAPSII: Simplified acute physiology score
SCCM: Society of critical care medicine
VAP: Ventilator-acquired pneumonia
VFD: Ventilator-free day
